# Dengue among Patients Visiting Department of Medicine in a Secondary Care Centre: A Descriptive Cross-sectional Study

**DOI:** 10.31729/jnma.8052

**Published:** 2023-07-30

**Authors:** Basanta Tamang, Ajaya Basnet, Sudip Khadka, Pramod Joshi, Roshan Kumar Jha, Rijuta Joshi, Shiba Kumar Rai

**Affiliations:** 1 Department of Clinical Laboratory, J and J Health Clinic, Ravibhawan, Kathmandu, Nepal; 2 Department of Medical Microbiology, Shi-Gan International College of Science and Technology, Shankhamarg, Kathmandu, Nepal; 3 Department of Microbiology and Immunology, Stanford University, Palo Alto, California, United States; 4 Department of Orthopaedic, National Trauma Center, Mahankal, Kathmandu, Nepal; 5 Department of Internal Medicine, Nepal Armed Police Force Hospital, Balambu, Kathmandu, Nepal; 6 Department of Gyneco-oncology, Patan Academy of Health Sciences, Lagankhel, Lalitpur, Nepal; 7 Research Division, Nepal Medical College and Teaching Hospital, Jorpati, Kathmandu, Nepal

**Keywords:** *biomarkers*, *dengue*, *Nepal*, *prevalence*, *prognosis*

## Abstract

**Introduction:**

Dengue, caused by the dengue virus, has a wide range of clinical features, including fever, body ache, lethargy, nausea, and vomiting. Blood-based biomarkers in patients with dengue virus infection reflect a variety of clinical spectrums, from bleeding manifestations to liver abnormalities, and can serve as an essential tool for clinicians. This study aimed to determine the prevalence of dengue among patients visiting the Outpatient Department in a secondary care centre.

**Method:**

A descriptive cross-sectional study was conducted among patients visiting the Outpatient Department from 16 May 2022 to 15 November 2022. Ethical approval was obtained from the Institutional Review Committee (Reference number: 20790202). The socio-demographic details and biochemical and haematological findings of dengue virus-infected patients diagnosed with rapid diagnostic tests were collected. Convenience sampling was done. Point estimate and 95% Confidence Interval were calculated.

**Results:**

Out of 706 individuals, the prevalence of dengue was 83 (11.76%) (9.38-14.14, 95% Confidence Interval). The median age of dengue virus-infected patients was 40 years, and the majority were males 54 (65.06%).

**Conclusions:**

The prevalence of dengue was found to be lower than in other studies done in similar settings.

## INTRODUCTION

Dengue is a mosquito-borne infection transmitted by the bite of infected blood-sucking female *Aedes aegypti* and *A. albopictus*.^1^ In Nepal, the first incidence of dengue virus (DENV) infection was reported in 2004, which was later followed by epidemics in 2006 (all four serotypes), 2010 (DEVN-1), 2013 (DEVN-2), and a yearly outbreak from 2014-2019.^2^

Identifying the group with minor illnesses from the ones progressing to potentially fatal conditions is always essential.^3,4^ Plasma leakage, with or without haemorrhage, is the hallmark pathological feature of dengue illness, accompanied by hepatic involvement that results in an alteration in blood biomarkers.^4^ Hence, unearthing dengue-specific laboratory determinants could early prognosticate the different phases of DENV illness and aid in obtaining a timely and accurate diagnosis of the patient.^3^

This study aimed to determine the prevalence of dengue among patients visiting the Outpatient Department of Medicine in a secondary care centre.

## METHODS

This descriptive cross-sectional study was conducted in the Outpatient Department of J and J Health Clinic, Ravibhawan, Kathmandu, Nepal, from 16 May 2022 to 15 November 2022. Ethical approval was taken from the Institutional Review Committee (Reference number: 20790202), Shankhamarg, Kathmandu, Nepal. The patients, aged 12-84 years, who had developed symptoms similar to DENV infection and had been detected with dengue using a lateral flow immunoassay (LFIA) approved by the Nepal Public Health Laboratory (US-FDA-EUA authorized) were taken as the study population. Patients who had visited the study in the time frame and were suspected of dengue were included in the study. Patients with incomplete study details but with a detection of DENV infection and patients with IgG positivity representing a past infection and not necessarily an ongoing infection were excluded from this study. Convenience sampling method was used. The sample size was calculated using the following formula:


$$\begin{array}{ll} \text{n} & ={\text{Z}}^{2}\times \frac{\text{p}\times \text{q}}{{\text{e}}^{\text{2}}}\\ & =1.96^{2}\times \frac{0.657\times 0.343}{0.05^{2}}\\ & =347 \end{array}$$

Where,

n = minimum required sample size
Z = 1.96 at 95% Confidence Interval (CI)
p = prevalence taken from a previous study as, 65.7%^5^
q = 1-p
e = margin of error, 5%

Hence, the minimum sample size required was 347. However, the final sample size taken was 706.

The patient information sheet recorded relevant demographic data and laboratory findings. Communication with the involved healthcare workers clarified any missing or ambiguous records. Participants informed consent was taken, and later, personal identifiers were removed.

Dengue was detected based on the measurement of IgM antibodies and NS1 levels. Briefly, 10 pl of the serum sample was added to the sample well "S" of the test device, which was immediately followed by adding one drop of the assay buffer solution to the same well. The result was interpreted after 10 minutes. Two distinct lines, one on the well "C" and one on the well "M", indicated the presence of IgM antibodies. Similarly, two distinct lines, one on well "C" and one on well "G", indicated the presence of IgG antibodies. Three distinct lines, one on well "C", one on well "G", and one on well "M", indicated the presence of both IgG and IgM antibodies developed against the dengue virus. Lastly, two distinct lines, one on well "C" and the other on well "NS1", indicated the presence of NS1 antigen. However, a single line on the mark "C" indicated a negative result. The absence of a distinct line in the control well indicated an invalid test. The positive control with distinct lines on the wells "C", "NS1", "IgM", and "IgG" and the negative control with one distinct line on well "C" were placed simultaneously.^6^

This study examined biochemical biomarkers, including total bilirubin (0.4-1.2mg/dL), direct bilirubin (0.0-0.4mg/dL), alanine transaminase (<37IU/L), aspartate aminotransferase (<42IU/L), alkaline phosphatase (108-306IU/L), and haematological parameters, including white blood cells (4,000-11,000/L), neutrophils (50-70%), lymphocytes (20-40%), monocytes (2-10%), eosinophils (1-6%), red blood cells (3.5-4.5 million/L), packed cell volume (33-42%) or hematocrit, mean corpuscular volume (76-96fl), mean corpuscular haemoglobin (26-36pg), mean corpuscular haemoglobin concentration (31-37gm/dL), haemoglobin (12-16gm/dL), and platelets (150,000-450,000/L). Since the laboratory reference values differ based on population, region, and institution, the reference values in this study were as per the institution guidelines. The biochemical biomarkers were analyzed by the ERBA CHEM-7 Biochemistry Analyzer (Germany), and the haematological biomarkers were analyzed by the Horiba Hematology Analyzer (Model: ABX Micros ES-60, USA).

Data was entered using Microsoft Excel 2010 and analysis was done using IBM SPSS Statistics version 17.0. Point estimate and 95% CI were calculated.

## RESULTS

Among 706 patients, dengue was detected in 83 (11.76%) (9.38-14.14, 95% CI) patients. The median age of dengue patients was 40 years and ranged from 12 to 84 years. Among the dengue patients, there were 5 (6.02%) children, 65 (78.31%) adults, and 13 (15.66%) elderly patients. (Table 1).

**Table 1:** Age and gender distribution dengue (n= 83).

Parameters			
Age group (years)	Male n (%)	Female n (%)	Total n (%)
15 years	4 (4.82)	1 (1.20)	5 (6.02)
16-59 years	40 (48.19)	25 (30.12)	65 (78.31)
60 years	10 (12.05)	3 (3.61)	13 (15.66)
Total	54 (65.06)	29 (34.94)	83 (100)

Among the total 706 individuals, 83 (11.76%) patients tested positive for dengue with NS1 detection, while 2 (0.28%) tested positive with IgM detection. Among 83 patients, 38 (45.78%) had ALT levels >37 IU/L, and 37 (44.58%) had AST levels >42 IU/L, both of which were higher than the reference range. Forty-seven (56.63%) patients had normal platelet count, and 36 (43.37%) patients had low platelet count (Table 2).

**Table 2:** Laboratory parameters among patients with dengue (n= 83).

Parameters	Laboratory values	n (%)
Alanine transaminase	>37 IU/L	38 (45.78)
Aspartate aminotransferase	>42 IU/L	37 (44.58)
Alkaline phosphatase	<108 IU/L	3 (3.61)
	>306 IU/L	4 (4.82)
Hemoglobin	<12 gm/dL	14 (16.87)
	>16 gm/dL	4 (4.82)
White blood cells	<4000/L	42 (50.60)
Neutrophil	<50%	8 (9.64)
	>70%	42 (50.60)
Lymphocyte	<20%	22 (26.51)
	>40%	9 (10.84)
Monocyte	<2%	25 (30.12)
Eosinophil	<1%	8 (9.64)
	>6%	6 (7.23)
Platelets	150,000/L	33 (39.76)
	>450,000/L	3 (3.61)
Red blood cells	3.5 million/L	2 (2.41)
	4.5 million/L	47 (56.63)
Hematocrit	<33%	5 (6.02)
	>42%	42 (50.60)
Mean corpuscular	<76 fl	5 (6.02)
volume	>96 fl	4 (4.82)
Mean corpuscular haemoglobin	<26 pg	8 (9.64)
Mean corpuscular haemoglobin concentration	<31 gm/dl	5 (6.02)
	>37 gm/dl	1 (1.20)

## DISCUSSION

Among 706 patients, dengue was detected in 83 (11.76%) patients. This finding was lower than in a similar study where the prevalence was found to be 65.7%.^5^ In a similar study conducted in Nepal, the prevalence was found to be 78.2% which was higher than in our study.^7^ The smaller sample size, corresponding to the lower generalizability, could attribute to such variation. Worldwide, Dengue inflicts a significant health burden, affecting more than 100 million individuals and resulting in 24,000 deaths annually.^5^

Among patients with dengue, 54 (65.52%) were males and 29 (34.48%) were females, with an approximate male-to-female ratio of 2:1. The sex ratio was comparable to the findings of several studies performed in India, with males being affected the most.^5^ The highest percentage of patients were in the age group of 16-59 years (adults) (78.31%), followed by the patients in the age group >60 years (elderly) (15.67%) and 15 years (children) (6.02%). Similarly, a study also showed a higher number of dengue infections in adults, specifically in the age groups of 21-30 years (49%) and 31-40 years (18.86%).^8^ Such a high prevalence of dengue in adults is because they form the working age group and have more exposure to insect bites.

However, the levels of the transaminases in adults (79.31%) and elderly (14.94%) in this study were within the reference range, unlike the findings of several studies reported elsewhere,^1,9,10^ which reported varying incidences of increase in ALT (17.6-39.2%) and AST (45.1-72.7%). Such an increment in serum AST could be responsible for the excess release of AST from damaged non-hepatic muscle cells (erythrocytes, kidney and brain tissue, cardiac and skeletal muscle) during dengue.^1^ ALT is mainly related with hepatocytes and is elevated because of damage to liver cells, indicating hepatic damage.^11^ Nevertheless, such increments show the degree of hepatocellular injury and suggest significant liver damage; therefore, they can be used as a potential marker to identify the disease.

In our study, leukocyte was observed in 26.51% of the patients, which was comparable with a study (>50%).^12^ However, a study reported a very high prevalence of leukopenia (94%) among patients with dengue.^13^ A study also reported leucopenia as one of the common heematological abnormalities.^14^ This study showed lymphocytopenia in 22 (26.51%) patients. In contrast, a study reported neutrophilia at the onset of the disease along with lymphocytosis, indicating an augmented immune response of the host to control the spread of dengue virus-infected cells.^15^

An overall prevalence of 33 (39.76%) of thrombocytopenia among patients with dengue infection was observed in this study. A study reported a very high incidence of thrombocytopenia (54%), including 38% of patients with mild thrombocytopenia (50-100/L), 14% with moderate thrombocytopenia (2050/L), and 2% with severe thrombocytopenia (20/L).^15^ Our findings of thrombocytopenia in patients with DENV infection were lower compared with several studies (54.70-81%).^4,8,16^ This could be because we assessed the blood samples of the study population at an early stage of dengue fever. Several studies have attributed bone marrow suppression by a virus, dengue antigens binding to platelets, increased immune-mediated destruction of platelets, and aggregation of platelets to virus-infected endothelium as possible causes for thrombocytopenia.^1,15^

In this study, low haemoglobin levels were observed in 14 (16.87%) patients, which was three times lower than the prevalence reported in a previous study (44.1%).^1^ Increased RBC count and hematocrit were observed in more than 50% of the patients in our study. Such an elevation in hematocrit, with varying ranges (6.9%-50%), was also observed in several studies.^1,8^ A study showed an increase in hematocrit level with increased disease severity and accredited such an increment to hemoconcentration due to increased intravascular plasma leakage.^1^

The limitations of this study were a smaller sample and a single-centred study, so this cannot be generalized to the whole population. Similarly, a consecutive follow-up of haematological and biochemical indices was not done, which could have provided a better understanding of the nature of disease progression. Regardless of the limitations, this study attempts to provide the prevalence in a healthcare setting and baseline data on the laboratory profiles of Nepalese patients with dengue, since there are limited studies in Nepal regarding the laboratory profiles of patients with dengue.

## CONCLUSIONS

The prevalence of dengue in this study was lower compared to similar studies done in similar settings. Lowered levels of leucocytes, monocytes, lymphocytes, and platelets and elevated levels of RBC, hematocrit, AST, and ALT are observed in patients with DENV infection. Identification of derangements in these biomarkers at the initial stages of dengue infection can facilitate early treatment.
